# INTERVENTIONS FOR CAREGIVERS AND PERSONS LIVING WITH DEMENTIA ACROSS THE LONG-TERM CARE CONTINUUM

**DOI:** 10.1093/geroni/igae098.0927

**Published:** 2024-12-31

**Authors:** Debra Dobbs, Hongdao Meng

**Affiliations:** Chair; Discussant

## Abstract

According to estimates from the Alzheimer’s Association 2023 Alzheimer’s disease Facts and Figures, there are an estimated 6.7 million people living with dementia (PLWD) in the United States. With increasing older age as the number one risk factor, this number will only continue to grow. Family and direct care workers (e.g., nurses, certified nursing assistants, social workers) provide the bulk of care to PLWD across the health care continuum. Interventions are needed to assist and improve dementia care for families, direct care workers and PLWD. This symposium includes four papers that have conducted dementia care interventions in different care settings. The first paper is a Palliative Care Education in Assisted Living intervention for nurses and administrators in 10 ALs to increase advance care planning documentation for AL residents (N=118) and to increase staff self-efficacy (N=23). Self-reported self-efficacy will be compared to treatment fidelity data on ACP discussions with family to examine how well the ACP discussion intervention protocol was adhered to. The second paper is an intervention study that employs mixed methods in a sample of 155 Veterans Health Administration clinicians to test the effectiveness of the Virtual Dementia Tour (VDT) and the impact the VDT has on their clinical practices. The third paper tested the feasibility of a group digital gaming intervention in a sample of 40 PLWD and their caregivers in adult day centers. The fourth paper tests the feasibility of an e-Pain support intervention for ADRD hospice patient caregivers. The scalability of each intervention will be discussed.

